# IMAGENE trial: multicenter, proof-of-concept, phase II study evaluating the efficacy and safety of combination therapy of niraparib with PD-1 inhibitor in solid cancer patients with homologous recombination repair genes mutation

**DOI:** 10.1186/s12885-022-10398-6

**Published:** 2022-12-09

**Authors:** Taigo Kato, Nobuaki Matsubara, Masaki Shiota, Masatoshi Eto, Takahiro Osawa, Takashige Abe, Nobuo Shinohara, Yota Yasumizu, Nobuyuki Tanaka, Mototsugu Oya, Koshiro Nishimoto, Takuji Hayashi, Masashi Nakayama, Takahiro Kojima, Kenjiro Namikawa, Takao Fujisawa, Susumu Okano, Eisuke Hida, Yoshiaki Nakamura, Hideaki Bando, Takayuki Yoshino, Norio Nonomura

**Affiliations:** 1 Department of Urology, Osaka University Graduate School of Medicine, 2-2 Yamadaoka, Suita, Osaka 565-0871 Japan; 2 Department of Medical Oncology, National Cancer Center Hospital East, Chiba, Japan; 3 Department of Urology, Graduate School of Medical Sciences, Kyushu University, Fukuoka, Japan; 4 Department of Urology, Hokkaido University Graduate School of Medicine, Hokkaido, Japan; 5 Department of Urology, Keio University School of Medicine, Tokyo, Japan; 6 Department of Uro-Oncology, Saitama Medical University International Medical Center, Saitama, Japan; 7 Department of Urology, Osaka International Cancer Institute, Osaka, Japan; 8 Department of Urology, Aichi Cancer Center, Aichi, Japan; 9 Department of Dermatologic Oncology, National Cancer Center Hospital, Tokyo, Japan; 10 Department of Head and Neck Medical Oncology, National Cancer Center Hospital East, Chiba, Japan; 11 Department of Biostatistics and Data Science, Osaka University Graduate School of Medicine, Osaka, Japan; 12 Department of Gastroenterology and Gastrointestinal Oncology, National Cancer Center Hospital East, Chiba, Japan; 13 Translational Research Support Section, National Cancer Center Hospital East, Chiba, Japan

**Keywords:** Homologous recombination repair, Niraparib, PARP inhibitor, Immune checkpoint inhibitor, Tumor-agnostic therapy

## Abstract

**Background:**

Previous clinical trials have demonstrated the potential efficacy of poly (ADP-ribose) polymerase (PARP) inhibitors (PARPis) in patients with cancer involving homologous recombination repair (HRR) gene-mutation. Moreover, HRR gene-mutated cancers are effectively treated with immune checkpoint inhibitors (ICIs) with the increase in tumor mutation burden. We have proposed to conduct a multicenter, single-arm phase II trial (IMAGENE trial) for evaluating the efficacy and safety of niraparib (PARPi) plus programmed cell death-1 inhibitor combination therapy in patients with HRR gene-mutated cancers who are refractory to ICIs therapy using a next generation sequencing-based circulating tumor DNA (ctDNA) and tumor tissue analysis.

**Methods:**

Key eligibility criteria for this trial includes HRR gene-mutated tumor determined by any cancer gene tests; progression after previous ICI treatment; and Eastern Cooperative Oncology Group Performance Status ≤ 1. The primary endpoint is the confirmed objective response rate (ORR) in all patients. The secondary endpoints include the confirmed ORR in patients with HRR gene-mutation of ctDNA using the Caris Assure (CARIS, USA). The target sample size of the IMAGENE trial is 57 patients. Biomarker analyses will be performed in parallel using the Caris Assure, proteome analysis, and T cell repertoire analysis to reveal tumor immunosurveillance in peripheral blood.

**Expected outcome:**

Our trial aims to confirm the clinical benefit of PARPi plus ICI combination therapy in ICI-resistant patients. Furthermore, through translational research, our trial will shed light on which patients would benefit from the targeted combination therapy for patients with HRR gene-mutated tumor even after the failure of ICIs.

**Trial registration:**

The IMAGENE trial: jRCT, Clinical trial no.: jRCT2051210120, Registered date: November 9, 2021.

## Background

Homologous recombination (HR) is a multifactorial process in DNA repair involving the repair of double strand brakes (DSB) generated at the broken replication forks [1]. In particular, HR repair (HRR) genes such as *BRCA1* and *BRCA2* play a critical role in carrying out successful HR. In normal cells, DSBs are repaired by the HRR mechanism to prevent cell death. By contrast, in cells with HRR dysfunction (HR deficiency: HRD), the damage DNA accumulates because of unsuccessful DSB, eventually leading to cell death [2]. These cells rely on DNA single-strand break repair such as base exchange repair (BER) mechanisms, including Poly (ADP-ribose) polymerase (PARP), an enzyme required for BER to maintain genomic stability [3]. PARP inhibitors (PARPis) induce synthetic lethality in cells with HRD and consequently exhibit antitumor effects. Taking this effect into account, PARPis have undergone clinical approval for the treatment of ovarian, breast and prostate cancers involving HRR gene mutations [4–10].

Recently, immune checkpoint inhibitors (ICIs) have been introduced with the aim to improve patient outcomes as evidenced by the encouraging results of several clinical trials [11–16]. Interestingly, HRR gene mutations are found in a particular patient subset in many types of cancer, and these patients are thought to have susceptibility to ICIs because HRR gene mutations generate immunogenic cancer-antigens owing to the accumulation of somatic mutations [17]. Indeed, several studies have shown that alterations in DNA damage response and repair genes are associated with response to programmed cell death 1/programmed cell death ligand 1 (PD-1/PD-L1) blockade in patients with several types of cancer [18, 19]. Considering above, we can expect synergistic effects of PARPi plus ICI combination therapy for patients with HRR-gene mutations.

The SCRUM-Japan consortium of the Nationwide Cancer Genome Screening Project with an industry-academia collaboration started the MONSTAR-SCREEN project, which evaluates the presence of circulating tumor DNA (ctDNA) before and after initiating cancer treatments [20, 21]. This ctDNA assay can reveal comprehensive somatic genomic alterations that enable the assessment of predominant spatial and temporal intratumoral heterogeneity with minimal invasiveness [22]. Utilizing this platform, we propose an investigator-initiated trial (IMAGENE trial) of niraparib (PARPi) and pembrolizumab or nivolumab combination therapy for HRR gene-mutated solid cancer based on the findings of ctDNA as well as tissue genome screening in eight institutions (sample size: 57, enrollment: 1 years and 8 months).

For personalized immunotherapy, we believe this tumor-agnostic trial would create scientific breakthrough that may lead to better treatments for patients with HRR-gene mutations.

## Methods

### Study design and treatment

**Fig. 1:**
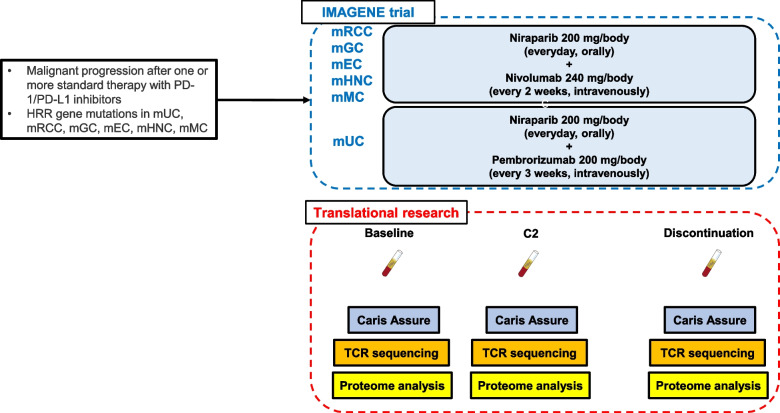
Overall trial design. As the translational research, liquid biopsies for the IMAGENE trial will be performed at baseline, cycle 2, and after discontinuation of protocol treatment. C2; Cycle 2, mEC; metastatic esophageal cancer, mGC; metastatic gastric cancer, mHNC; metastatic head and neck cancer, mMC; metastatic melanoma, mRCC; metastatic renal cell carcinoma, mUC; metastatic urothelial carcinoma, PD-1; programmed cell death-1, PD-L1; programmed cell death ligand-1, TCR; T cell receptor

The present trial is a multicenter, single-arm, proof-of-concept (POC), phase II study. aimed to evaluate the efficacy and safety of combination therapy of niraparib plus nivolumab or pembrolizumab (PD-1 inhibitor) in patients with solid cancer involving HRR gene mutations. This study is conducted as a tumor-agnostic trial for patients with metastatic urothelial carcinoma (mUC), metastatic renal cell carcinoma (mRCC), metastatic gastric cancer (mGC), metastatic esophageal cancer (mEC), metastatic head and neck cancer (mHNC), and metastatic melanoma (mMC). HRR gene mutations were determined by tumor tissue analysis including FoundationOne CDx, OncoGuide NCC oncopanel, and Caris Molecular Profiling (Fig. 1). In this study, FoundationOne liquid CDx, Guardant 360, and Caris Assure are also used to screen patients harboring HRR gene mutations using ctDNA tests in blood samples (Fig. 1). The HRR genes are defined as *BRCA1*, *BRCA2*, *ATM*, *CDK12*, *CHEK2*, *PALB2*, *BARD1*, *BRIP1*, *CHEK1*, *FANCL*, *RAD51B*, *RAD51C*, *RAD51D*, and *RAD54L*. This trial has been registered in the Japan Registry of Clinical Trials (jRCT2051210120).

### Patients

The eligibility criteria for this trial are presented in Table 1. Key eligibility criteria include the presence of HRR gene mutated tumors determined by an analysis of the tumor tissue or blood sample; tumor progression after treatment with a previous PD-1 or PD-L1 inhibitor; and Eastern Cooperative Oncology Group Performance Status score of ≤ 1. All patients are enrolled in the molecular profiling study, SCRUM-Japan MONSTAR-SCREEN-2 (UMIN000043899), which incorporate a tissue (Caris Molecular Profiling) and plasma multiomics approach (Caris Assure) based on artificial intelligence. Enrollment has started on April 2022 and will be completed on August 2023.

**Table 1:** Eligibility criteria for the IMAGENE trial

Inclusion criteria	Exclusion criteria
1. Malignant progression after one or more standard therapy with PD-1/PD-L1 inhibitors, and completion of standard therapy in principle	1. Severe comorbidity a. Synchronous active malignancies b. Uncontrolled brain metastasis or leptomeningeal metastasis c. Active infectious disease d. Uncontrolled ascites, pleural effusion, or pericardial effusion requiring continued drainage e. Uncontrolled diabetes mellitus or hypertension f. Myocardial infarction, severe/unstable angina pectoris, symptomatic congestive heart failure of New York Heart Association Class II—IV within 6 months before enrollment g. Psychiatric diseases or psychiatric symptoms that were considered to cause difficulty in enrollment in a clinical trial
2. Diagnosed with the target disease (unresectable advanced or recurrent urothelial cancer, renal cell carcinoma, gastric cancer, esophageal cancer, head and neck cancer, malignant melanoma with HRR gene mutation in the germline or somatic cells, which were detected by the specified tests)	2. Underwent one of the following treatments before protocol treatment: a. Extensive surgery within 4 weeks b. Colostomy/ileostomy within 2 weeks c. Chemotherapy within 2 weeks d. Radiation therapy within 2 weeks
3. Measurable lesions	3. CTCAE Grade 2 adverse events due to previous therapy, which are not recovered
4. Legal adult on informed consent	4. History of PARP inhibitor treatments
5. ECOG PS: 0 or 1	5. Intolerant to previous irinotecan therapy
6. Appropriate physical function confirmed by laboratory values within 14 days before enrollment	6. Comorbidity or history of uncontrollable hypertension
7. (Female of childbearing potential) Agreed to contraception and not to donate oocytes from consent to 180 days after the last dose of the study drug	7. Men/women who are unwilling to avoid pregnancy; women who are pregnant or breastfeeding; women with a positive pregnancy test
8. (Male) Agreed to contraception from the start of study treatment to 180 days after the last dose of the study drug, and agreed not to donate sperms from the start of study treatment to 90 days after the last dose of the study drug or 120 days after the last dose of a PD-1 inhibitor, whichever is later	8. Known active HCV or HIV infection
9. Written consent to participate in the clinical trial	9. Any other patients who are regarded as inadequate for trial enrollment by investigators

In addition to this clinical trial, patients who do not meet all inclusion criteria, or those meet any of the exclusion criteria are enrolled in the natural history follow-up cohort and will be observed on follow up to collect information on anti-tumor treatment, post-treatment and survival every 3 months as a historical control (SCRUM-Japan registry, UMIN000028058).

### Treatments

Patients with mRCC, mGC, mEC, mHNC, and mMC will be treated with niraparib 200 mg daily and nivolumab 3 mg/kg every 2 weeks, whereas those with mUC will be treated with niraparib 200 mg daily and pembrolizumab 3 mg/kg every 3 weeks. Patients will receive therapy until reaching either disease progression or unacceptable toxicity. The dose of niraparib can be reduced to 200 mg daily according to adverse events during the treatment.

### Endpoints and statistical analysis

The primary endpoint is the objective response rate (ORR) confirmed by the investigators' assessment in patients with HRR genes mutation determined by any cancer gene panel. The secondary endpoints include the confirmed ORR in patients with HRR gene mutations in ctDNA determined by Caris Assure, progression-free survival (PFS), overall survival (OS), disease control rate (DCR), duration of response (DOR), time to treatment failure (TTF), and change rate of tumor size, as determined by the investigators' assessment, and the incidence of adverse events (AEs). Efficacy will be evaluated according to Response Evaluation Criteria in Solid Tumors (RECIST) V.1.1 using computed tomography every 6 weeks until the end of cycle 12 (nivolumab) or 8 (pembrolizumab), and thereafter every 8 weeks. AEs are assessed according to the Common Terminology Criteria for Adverse Events V.4.0 before the administration of the investigational drug on the day of administration. In this trial, the threshold of the ORR has been set to 16%, and the expected rate to 31%, as niraparib combined with ICIs based on the results of previous clinical trials of ICIs. Under this assumption, with a one-sided significance level of 5% and a power of 80%, the sample size required was calculated to be 57. The sample size for each tumor was set at 15 for mUC, 4 for mRCC, 15 for mGC, 8 for mEC, 13 for mHNC and 2 for mMC, based on feasibility and the expected proportion of participants.

All analysis will be based on the intention-to-treat principle. The baseline characteristics will be described as the mean and standard deviation, or median and quantiles (for continuous variables), or proportion (for categorical variables). The binomial test will be used to analyze the primary endpoint ORR and a threshold, and the Clopper-Pearson method will use the 95% confidence interval for ORR and DOR. Estimates of PFS, DCR, DOR, TTF, and OS will be used with the Kaplan–Meier method.

### Biomarker analyses and translational research

ctDNA analysis using a targeted next-generation sequencing (Caris Assure) will be performed at baseline, Cycle 2, and after treatment discontinuation. We will also perform three types of analysis (namely, PD-L1 expression analysis, next-generation sequencing-based T cell repertoire analysis, and quantitative proteomics analysis) to investigate predictive biomarkers of response to this combination therapy. First, the expression level of PD-L1, a well-known biomarker of the clinical response to ICIs in cancer tissues, will be evaluated. Second, we will perform T cell repertoire analysis based on next-generation sequencing to determine whether T cell receptor (TCR) diversity in the peripheral blood can serve as a useful indicator of response to combination therapy. Third, we will perform a mass spectrometry-based quantitative proteomic analysis using plasma to identify candidate proteins as potential predictive biomarkers.

## Discussion

The introduction of next-generation sequencing technologies and large-scale tumor molecular profiling tests have revolutionized the field of precision oncology [23]. Precision oncology has already transformed cancer treatment for both common and rare malignancies that are targeted by specific therapies to improve clinical outcomes in patients. HRR gene-mutated tumor are one of the rare fractions across cancers, which are reported to be approximately 3% – 20% [24–29], and require more effective treatment beyond PARPi monotherapy. In this regard, our trial will reveal some important points for selecting patients who benefit from a PARPi in combination with ICIs as tumor-agnostic therapy.

First, our trial could first reveal the real benefit of PARPi plus ICI combination therapy for patients who are refractory to previous treatment with ICIs for a subset of HRR gene-mutated tumors. Importantly, PARPis are compatible with ICIs for three reasons: 1) PARPis upregulate PD-L1 expression in cancer cells in vitro and in vivo which is a marker for response to ICIs; 2) PARPis release DNA from cancer cells and induce T cell activation through the release of interferon-γ by the activation of the STING pathway; and 3) PARPis change the tumor microenvironment to activate tumor-infiltrating lymphocytes. Considering these possible benefits, our trial could reveal additional effects of PARPi combined with ICI in ICIs-resistant cancers [17, 30].

Second, our trial could show whether patients with HRR gene-mutated solid tumors identified by ctDNA genotyping benefit from PARPi plus ICI similarly to patients identified by conventional tissue analysis. To date, only one study from our group has reported that pertuzumab plus trastuzumab showed similar efficacy in patients with mCRC with HER2 amplification in ctDNA and tumor tissues [31]. Considering that ctDNA may offer clinical advantages for assessing tumor heterogeneity associated with acquired resistance, our trial could contribute to the critical assessment for further use of ctDNA genotyping clinical trials.

Third, our trial results will help perform translational research to decipher the real benefit from PARPi plus ICI combination therapy and could clarify the dynamics of tumor immunosurveillance in both tissues and peripheral blood in our setting. We previously reported that peripheral TCR analysis and early ctDNA dynamics could predict clinical response to ICIs [32]. Franz et al. reported that the clinical utility of proteomic analysis may clarify the responders to PAPRi [33]. Given the additional effect of PAPRi for modulating the tumor microenvironment and cancer-specific T cells, our combined novel analysis could identify the subgroup that shows a good response to this combination therapy.

In conclusion, to the best of our knowledge, the IMAGENE trial is the first phase II study to evaluate the efficacy, safety and POC of combination therapy with niraparib and ICI in patients with HRR gene-mutated tumor. For precision oncology, these findings will shed light on the potential value of targeted combination therapy for patients with HRR gene-mutated tumors even after the failure of ICIs treatment.

## Data Availability

Not applicable.

## Abbreviations

CTCAE: Common Terminology Criteria for Adverse Event
ICI: Immune checkpoint inhibitor
ORR: Objective response rate
OS: Overall survival
PARPi: Poly (ADP-ribose) polymerase inhibitor
PD-1: Programmed cell death-1
PD-L1: Programmed cell death ligand-1
PFS: Progression-free survival
